# Commentary: A new gadget for redo tricuspid surgery

**DOI:** 10.1016/j.xjtc.2022.02.042

**Published:** 2022-04-11

**Authors:** Min Ho Ju, Joon Bum Kim

**Affiliations:** a Department of Cardiovascular and Thoracic Surgery, Research Institute for Convergence of Biomedical Science and Technology, Pusan National University - Yangsan Hospital, Yangsan-Si, South Korea; b Department of Thoracic and Cardiovascular Surgery, Asan Medical Center, University of Ulsan College of Medicine, Seoul, South Korea

**Figure:**
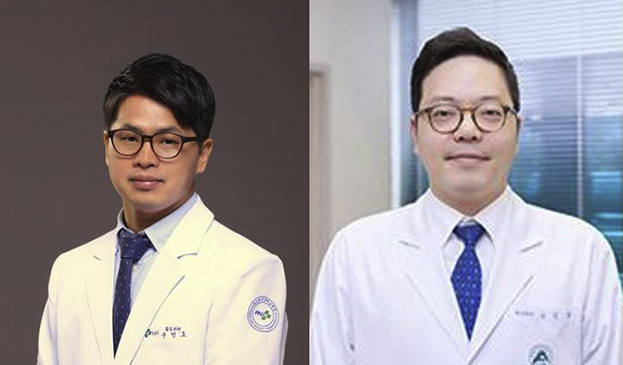
Min Ho Ju, MD, and Joon Bum Kim, MD, PhD

**Central Message**

It is always good to have many tools, and with the addition of this new gadget, we may be on more solid footing to deal with various challenging cases of TV surgery.

See Article page 62.

In the setting of redo isolated tricuspid valve (TV) surgery, a right thoracotomy approach is a reasonable alternative to redo sternotomy, by which time consumption required to dissect into the pericardial space can be reduced while injury to the heart and great vessels during the adhesiolysis can also be minimized.^1^^,^^2^ As isolated TV surgery can also be performed under beating heart without a need of aorta manipulation, the so-called “minimalist approach” of performing TV surgery under beating heart using a minithoractomy and percutaneous peripheral cannulations has been popularized by leading experts in recent years.^2^ For a more reproducible execution of this procedure, it is important to secure effective venous drainage so that venous blood does not interfere with exposing TV. In addition, venous drainage should not be interrupted by air trapping in the draining cannulae.

In this issue of *JTCVS Techniques*, Bitargil and colleagues^3^ introduce a method of implementing a caval isolation technique using a commercially available balloon catheter. In a challenging case of severe tricuspid regurgitation following heart transplantation undergoing TV replacement under a right thoracotomy approach and peripheral cannulations, the authors used a very ordinary medical instrument—the Fogarty catheter, which was introduced within the lumen of caval cannulae to effectively block the blood from entering the right heart. Without such a method, isolation and occlusion of the venae cavae might have required extensive adhesiolysis, which is challenging in the heart transplantation recipient. The method introduced by the authors seems simple and effective without a need for additional invasive procedure, by which it may serve as one alternative gadget in dealing with similar cases.

Among up-to-date minimally invasive cardiac surgeons, however, a more preferable method may be vacuum-assisted venous drain over the bicaval occluding method, like in the cited paper by Bitargil and colleagues in minithoracotomy TV surgery. In this percutaneous approach, venous cannulations may involve either bicaval cannulation (via both jugular and femoral) or even a single femoral cannulation but without occluding the draining site, allowing vacuum-assisted open draining of blood entering right atrium, which offers excellent exposure of the TV. The efficacy and efficiency of the vacuum-assisted venous drain in TV surgery were well demonstrated by clinical data, and this approach will be the benchmark of the technique presented by Bitargil and colleagues.^4^ One scenario for which this new technique may be preferable perhaps is time-consuming heavy procedures, in which clinically significant hemolysis may occur attributed to a long duration of vacuum suctioning of blood in open chamber. In such case, this new technique will offer safer room to run the pump without such a concern. As such, it is always good to have many tools available to handle a wide variety of technical issues, and with the addition of this new gadget, we may be on a more solid foothold to deal with various challenging cases of TV surgery.
